# *Euglena gracilis* Protein: Effects of Different Acidic and Alkaline Environments on Structural Characteristics and Functional Properties

**DOI:** 10.3390/foods13132050

**Published:** 2024-06-27

**Authors:** Laijing Zhu, Meng Liu, Yanli Wang, Zhunyao Zhu, Xiangzhong Zhao

**Affiliations:** School of Food Science and Engineering, Qilu University of Technology (Shandong Academy of Sciences), Jinan 250353, China; 15725152458@163.com (L.Z.); 15864530661@163.com (M.L.); 15106614358@163.com (Y.W.); 18366260356@163.com (Z.Z.)

**Keywords:** *Euglena gracilis* protein, structural characteristics, functional properties, acidic and alkaline environments

## Abstract

Due to the growing demand for human-edible protein sources, microalgae are recognized as an economically viable alternative source of proteins. The investigation into the structural characteristics and functional properties of microalgin is highly significant for its potential application in the food industry as an alternative source of protein. In this research, we extracted protein from *Euglena gracilis* by using alkaline extraction and acid precipitation and investigated its structural characteristics and functional properties in different acidic and alkaline environments. The molecular weight distribution of *Euglena gracilis* protein (EGP), as revealed by the size exclusion chromatography results, ranges from 152 to 5.7 kDa. EGP was found to be rich in hydrophobic amino acids and essential amino acids. Fourier infrared analysis revealed that EGP exhibited higher α-helix structure content and lower β-sheet structure content in alkaline environments compared with acidic ones. EGP exhibited higher foaming properties, emulsifying activity index, solubility, free sulfhydryl, and total sulfhydryl in pH environments far from its isoelectric point, and lower fluorescence intensity (2325 A.U.), lower surface hydrophobicity, larger average particle size (25.13 µm), higher emulsifying stability index, and water-holding capacity in pH environments near its isoelectric point. In addition, X-ray diffraction (XRD) patterns indicated that different acidic and alkaline environments lead to reductions in the crystal size and crystallinity of EGP. EGP exhibited high denaturation temperature (T_d_; 99.32 °C) and high enthalpy (ΔH; 146.33 J/g) at pH 11.0, as shown by the differential scanning calorimetry (DSC) results. The findings from our studies on EGP in different acidic and alkaline environments provide a data basis for its potential commercial utilization as a food ingredient in products such as emulsions, gels, and foams.

## 1. Introduction

The use of microalgae as a rich source of bioactive compounds such as proteins, polysaccharides, and polyunsaturated fats has garnered significant scientific attention in recent years [1]. Their consumption has been historically documented in human diets for thousands of years, and microalgae continue to be traditional dietary components in some areas [2]. With the increasing global need for protein resources, microalgae have emerged as a promising bulk protein source [3], as they entail low cultivation costs, grow rapidly, and do not encroach on terrestrial food production [4]. Furthermore, multiple microalgae can serve as a superior protein substitute due to high total protein contents and balanced essential amino acid (EAA) components compared with more traditional protein sources [3]. Many traditional foods made from microalgae, such as biscuits, ice cream, and gelled foods, not only present desirable flavor characteristics but also offer high nutritional value and contribute to a healthy diet [5]. Therefore, it is crucial to investigate the structural characteristics and functional properties of microalgal proteins in order to address the global protein shortage and provide essential data for the commercialization of microalgal proteins as a novel food ingredient. Species of microalgae of the genus *Euglena* are significant sources of a diverse range of bioactive compounds, such as protein, carotenoids, polyunsaturated fatty acids, and other bioactive compounds with antioxidant and anti-inflammatory properties, and have the potential to be utilized as novel foods [6]. *Euglena gracilis*, belonging to *Euglena* of the phylum *Euglenophyta*, serves as an excellent protein source, as its protein content is approximately 39–60% [3], containing a rich array of amino acids [7]. Furthermore, animal and in vitro studies have shown excellent digestibility of protein derived from *Euglena gracilis* [7].

The extraction method, pH, and other factors all exert an influence on the functional properties of proteins [8]. Varied acidic and alkaline environments can impact their structural characteristics and functional properties, thereby broadening the potential applications of proteins in the food industry. Benelhadj et al. [5] observed that Arthrospira (*Spirulina*) platensis protein isolate exhibits higher solubility, foaming capacity, and emulsifying capacity in alkaline environments. The solubility, emulsifying properties, foaming properties, and water-holding capacity of *Haematococcus pluvialis* protein were significantly improved after pH treatment [9]. Min-Soo Jeong et al. [10] reported that alkaline pH treatment reduced the β-sheet structure content in mung bean protein isolate, which exhibited a flexible structure along with excellent water-holding capacity (WHC) and gelation properties at pH 12.0. To fully utilize these proteins in the food industry, it is essential to investigate their structural characteristics and functional properties in different acidic and alkaline environments.

So far, limited research regarding the properties of *Euglena gracilis* protein (EGP), especially its structural characteristics and functional properties, has been published [11]. Therefore, the objective of our experiment was to extract EGP from *Euglena gracilis* and investigate the impact of the pH environment on its functional properties and structural characteristics by selecting pH values of 3.0, 5.0, 7.0, 9.0, and 11.0, representing different acidic and alkaline environments commonly encountered in various food systems. More in-depth research on the structural characteristics and functional properties of EGP in different acidic and alkaline environments will contribute to the basis for its application as a food ingredient in the food industry.

## 2. Materials and Methods

*Euglena gracilis* powder was obtained from Guangyu Biological Technology Co., Ltd. (Shanghai, China). Sodium hydroxide (NaOH), hydrochloric acid (HCl), and potassium bromide (KBr) were purchased from Sinopharm Group Co., Ltd. (Shanghai, China). All other analytical reagents were obtained from Sigma-Aldrich Co., Ltd. (St. Louis, MO, USA). Bovine serum albumin (of HPLC grade) was purchased from Solarbio Co., Ltd. (Beijing, China); peroxidase (of HPLC grade) from Innochem Co., Ltd. (Beijing, China); myoglobin and cytochrome C (of HPLC grade) from Aladdin Co., Ltd. (Shanghai, China); and aprotinin (of HPLC grade) from Yuanye Biotechnology Co., Ltd. (Shanghai, China). Soybean oil was obtained from a Jiajiayue supermarket (Jinan, China).

### 2.1. Preparation of Euglena gracilis Protein

*Euglena gracilis* protein was extracted by referring to the method by Zhong et al. [12] with slight amendments. The *Euglena gracilis* powder was dispersed in deionized water (1:60, *w*/*v*, g/mL), and the obtained dispersion was agitated using the overhead agitator (DLAB Scientific Co.Ltd., DLAB OS20/40-Pro; Beijing, China) in a 60 °C water bath for 2 h after adjusting the pH to 11.5 with 1 M NaOH and then centrifuged at 10,000× *g* for 20 min. The collected supernatant was centrifuged at 10,000× *g* for 20 min after adjusting the pH to 4.5 (isoelectric point) with 1 M HCl. The obtained precipitate was redispersed in deionized water; then, the dispersion pH was adjusted to 7.0 with 1 M NaOH. The collected dispersion was lyophilized (Xinzhi Biotechnology Co., Ltd., Scientz-18 N; Ningbo, China) after dialyzing with a dialysis bag at 4 °C for 48 h. The determined EGP purity according to the Kjeldahl method [9] was 85.27% (N × 6.25).

### 2.2. Molecular Weight Distribution (MWD)

Size exclusion chromatography (SEC), as described by Huang et al. [13] with slight amendments, was employed to determine the molecular weight distribution of EGP by using a Shimadzu liquid chromatograph (Shimadzu Corporation, Kyoto, Japan) with a 220 nm UV detector. The experiment was performed by using a TSKgel G2000SWxl column (Tosoh Bioscience, Kyoto, Japan), which was calibrated with a standard protein solution between 67 and 6.5 kDa (bovine serum albumin: 67 kDa; peroxidase: 40.2 kDa; myoglobin: 17 kDa; cytochrome C: 12.4 kDa; aprotinin: 6.5 kDa). The lyophilized EGP samples under different pH treatments were dispersed in the mobile phase to obtain a solution (0.2 mg/mL), followed by filtration through a filter head (0.22 µm). The sample injection volume and the flow rate were 10 μL and 1 mL/min, respectively. The mobile phase composition was phosphate buffer (0.05 M; pH 7.0) containing NaCl (0.3 M).

### 2.3. Amino Acid Composition

A total of 50 mg of EGP was dispersed in a 4 mL 6 M HCl decomposition tube to be hydrolyzed at 110 °C for 24 h after nitrogen blowing for 15 min. The obtained hydrolysate was diluted in a 100 mL volumetric flask, from which 2 mL was taken for deacidification at 60 °C until dry. After 0.02 mol/L HCL was added and mixed, the obtained mixture was filtered with a filter column (0.22 μm) and tested with an amino acid analyzer (Biochrom Co., Ltd., Biochrom 30+; Cambridge, UK) (Zhu et al. [9]).

### 2.4. Fourier Transform Infrared Spectroscopy (FTIR) of EGP

As described by He et al. [14] with slight amendments, lyophilized EGP samples (5.0 mg) under different pH treatments and with potassium bromide (KBr) (100 mg) (Sigma-Aldrich Company, St. Louis, MO, USA) were pressed into thin slices. The processed thin sections obtained were analyzed by using a Fourier transform infrared spectrometer (Thermo Fisher Scientific Co., Ltd., Nicolet iS20; Waltham, MA, USA) over a range of 4000 to 400 cm^−1^, and 32 scans were performed for each sample at ambient temperature with a resolution of 4 cm^−1^. The secondary structure of EGP was analyzed by using Peakfit ver. 4.12 software.

### 2.5. Foaming Properties

Referring to the method by Malomo and Aluko [15] with minor amendments, the lyophilized EGP samples were dispersed in deionized water to obtain dispersions (10 mg/mL), whose pH was adjusted to 3.0–12.0. The dispersions were homogenized at 10,000 rpm for 2 min at ambient temperature, and the foam volume of the dispersions was documented at 0 min and 30 min. FC and FS were determined by using the following formulas (Equations (1) and (2)):

(1) $$\mathrm{FC}(\%)=\left(V_{0}/V\right)\times 100$$

(2) $$\mathrm{FS}(\%)=\left(V_{1}{/V}_{0}\right)\times 100$$

In the above, V_0_ (mL), V_1_ (mL), and V (mL) represent the foam volume at 0 min and 30 min and the initial volume, respectively.

### 2.6. Emulsifying Properties

According to the procedure by Chee et al. [16] with minor amendments, aliquots of the lyophilized EGP samples were dispersed in deionized water to obtain dispersions (1 mg/mL), whose pH was adjusted to 3.0–12.0. The aliquot-based dispersions (21 mL) and soybean oil (7 mL) were homogenized (IKA Company, T25; Staufen, Germany) at 10,000 rpm for 2 min at ambient temperature. After homogenization, we immediately pipetted 100 μL from the bottom of the emulsion into a 25 mL tube and diluted it 100 times with 0.1% SDS (10 mL). The absorbance of the mixed, diluted emulsions was determined at 500 nm versus a 0.1% SDS blank by using a spectrophotometer (Yipu Instrument Co., Ltd., XU-8; Shanghai, China). We repeated the same procedure after 10 min to measure the absorbance of the mixed dilution emulsions. The EAI and ESI were computed by referring to Formulas (3) and (4).

(3) $$\mathrm{EAI}\left(m^{2}/g\right)=\left(2\times 2{.303\times A}_{0}\times \mathrm{DF}\right)/\left(c\times \varphi \times 10000\right)$$

(4) $$\mathrm{ESI}\left(\min\right)=\left[{\mathrm{EAI}}_{0\min}/\left({\mathrm{EAI}}_{0\min\;}-{\mathrm{EAI}}_{10\min}\right)\right]$$

In the above, A_0_ and A_10_ represent the absorbance values of the diluted emulsion at 0 min and 10 min, respectively. DF represents the dilution factor (100), c (g/mL) represents the concentration of the EGP dispersion, φ (0.25) represents the volume fraction of oil, and EAI_0min_ and EAI_10min_ represent the absorbance values of the diluted emulsion at 0 min and 10 min, respectively.

### 2.7. Solubility

As described by Lawal et al. [17], an aliquot (100 mg) of EGP was dissolved in deionized water. The dispersion (10 mg/mL) was agitated for 30 min after adjusting the pH to 3.0–12.0, followed by centrifugation at 6000× *g* for 15 min at ambient temperature. The protein content of the obtained supernatant and the total protein content were determined by using the Bradford method and the Kjeldahl method, respectively. The solubility of EGP was computed as follows:

(5) $$\mathrm{Solubility}(\%)=\left(\mathrm{protein}\;\mathrm{in}\;\mathrm{the}\;\mathrm{supernatant}/\mathrm{total}\;\mathrm{protein}\right)\times 100$$

### 2.8. Free and Total Sulfhydryl

The Ellman’s reagent method, as described by Plazzotta et al. [18], was employed with minor amendments to determine the SH content of EGP. The lyophilized EGP samples were dispersed in deionized water to obtain dispersions (10 mg/mL), whose pH was adjusted to 3.0–12.0. The DTNB (5,5′-dithiobis-2-nitrobenzoic acid) was dispersed into a tris-glycine buffer (0.086 M Tris, 0.090 M Gly, and 0.004 M EDTA; pH 8.0) to obtain Ellman’s reagent (4 mg/mL). Ellman’s reagent (50 μL), tris-glycine buffer (2 mL), and EGP dispersion (0.5 mL) were mixed rapidly. The obtained mixture was incubated in a dark environment for 30 min, and the absorbance was measured by using a UV–visible spectrophotometer (Yipu Instrument Co., Ltd., XU-8; Shanghai, China) at 412 nm. Triglycine buffer was combined with Ellmann’s reagent in a ratio of 40:1 to serve as the blank, wherein the sample solution was substituted with deionized water. The determination procedure for total sulfhydryl group content is identical to that for free sulfhydryl group content, with the exception that tris-glycine buffer contains urea (8 M) [19]. The content of free sulfhydryl was computed by using Formula (6).

(6) $$\mathrm{SH}\left(\mu \mathrm{mol}/g\right)=\left(73{.53\times A}_{412}\times D\right)/C$$

In the above, A_412_ stands for the absorbance at 412 nm, C stands for the EGP dispersion concentration (mg/mL), D stands for the dilution factor (1), and 73.53 was based on 10^6^/1.36 × 10^−4^, where 1.36 × 10^−4^ is the molar absorbance of Ellman’s reagent [20].

### 2.9. Water-Holding Capacity (WHC)

The WHC of EGP was measured by referring to the previous study by Abarghoei et al. [21]. Aliquots (500 mg) of EGP were added to deionized water (10 mL) in pre-weighted tubes, and the mixtures’ pH was adjusted to 3.0–12.0. After equilibrating for 30 min, the mixtures were centrifuged at 8000× *g* for 15 min at ambient temperature. The precipitate was weighted, and WHC was computed by using Formula (7).

(7) $$\mathrm{WHC}\left(g/g\right)=\left(W_{2}{-W}_{1}\right){/W}_{0}$$

In the above, W_0_ (g) represents the mass of EGP, W_1_ (g) represents the mass of EGP plus the centrifuge tube, and W_2_ (g) represents the mass of the precipitate plus the plastic centrifuge tube.

### 2.10. Fat Absorption Capacity (FAC)

The FAC of EGP was analyzed by using the procedure by He et al. [22] with a slight modification. EGP aliquots (500 mg) were weighted in pre-weighted centrifuge tubes and thoroughly mixed with 5 mL of soybean oil, followed by processing in a water bath at 30, 40, 50, 60, 70, 80, 90, and 100 °C for 30 min. After equilibration, the mixture was centrifuged at 8000× *g* for 10 min to separate the unbound water and the precipitate was weighed. The FAC was determined as follows:

(8) $$\mathrm{FAC}(\left(g/g\right)=\left(F_{2}{-F}_{1}\right){/F}_{0}$$

In the above, F_0_ stands for the mass of EGP (g), F_1_ stands for the mass of the tube plus EGP (g), and F_2_ stands for the mass of the tube plus the precipitate (g)

### 2.11. Particle Size Distributions

Based on earlier research by Jiang et al. [23], EGP (50 mg) was dispersed in deionized water (10 mL) to obtain dispersions. The pH of the EGP dispersions (5 mg/mL) was adjusted to 3.0, 5.0, 7.0, 9.0, and 11.0, and the particle size distribution was measured at ambient temperature by using a particle size and shape analyzer (Microtrac Retsch Gmbh Company, Microtrac MRB sync; Dusseldorf, Germany).

### 2.12. Zeta Potential

EGP (50 mg) was dispersed in deionized water (10 mL) to obtain dispersions. The pH of the EGP dispersions (5 mg/mL) was adjusted to pH 3.0, 5.0, 7.0, 9.0, and 11.0, and the zeta potential was determined at ambient temperature by using a laser particle size analyzer (Malvern Instruments Co., Ltd., Zetasizer Nano ZS90; Worcestershire, UK) (Gundogan et al. [24]).

### 2.13. Exogenous Fluorescence Spectroscopy

Aliquots (4 mL) of EGP dispersion (0.1 mg/mL) with pH adjusted to 3.0, 5.0, 7.0, 9.0, and 11.0 were combined with 20 µL of ANS (0.008 M) solution. The obtained mixtures were examined by using a fluorescence spectrophotometer (Hitachi.,Ltd., Hitachi F2700; Tokyo, Japan) to assess surface hydrophobicity. The scanning wavelength range, excitation wavelength, and slit width were set to 394–728 nm, 390 nm, and 5 nm, respectively (Ai et al. [25])

### 2.14. Intrinsic Fluorescence Spectroscopy

A fluorescence spectrometer (Hitachi.,Ltd., Hitachi F2700; Tokyo, Japan) was used to analyze the tertiary structure of EGP, as outlined by Xu et al. [26]. Briefly, aliquots (3 mL) of EGP dispersion (0.003 mg/mL) with pH adjusted to 3.0, 5.0, 7.0, 9.0, and 11.0 were scanned in the range of 300–540 nm. The excitation wavelength and slit width were set to 280 nm and 10 nm, respectively.

### 2.15. X-ray Diffraction (XRD)

X-ray diffraction analysis was employed as conducted by Ma et al. [27] with slight modifications. We spread the lyophilized EGP samples under different pH treatments on the test rack and then used an X-ray diffractometer (Bruker Corporation, Bruker D8 Advance; Saarbrucken, Germany) with Cu target anode material as the radiation source. The scanning rate was set to 5°/min, and the scanning range was from 5 to 90°.

### 2.16. Thermal Properties

A differential scanning calorimeter (Mettler-Toledo International Inc., Mettler DSC3; Zurich, Switzerland) was utilized to analyze the thermal properties of EGP by referring to the procedure by Kaushik et al. [28]. Briefly, EGP samples (around 5 mg) under different pH treatments were placed in a hermetically sealed aluminum pan. The calorimetry analysis of EGP was performed at a heating rate of 10 °C/min from 0 to 200 °C, with an empty aluminum pan serving as a blank.

### 2.17. Statistical Analysis

All experimental results were expressed as means ± standard deviation of three repeated measurements. Statistical analyses were carried out to generate graphs by using Origin 2021 9.8 software (OriginLab Corporation; Northampton, MA, USA) and Graphed Prism 10.1.2 (GraphPad Software Corporation; Boston, MA, USA). Significant differences (*p* < 0.05) were analyzed by using analysis of variance (ANOVA) with IBM SPSS Products:Statistics Common 26.0.0.0.0 (SPSS Inc.; Chicago, IL, USA).

## 3. Results

### 3.1. Molecular Weight Distribution

**Figure 1:**
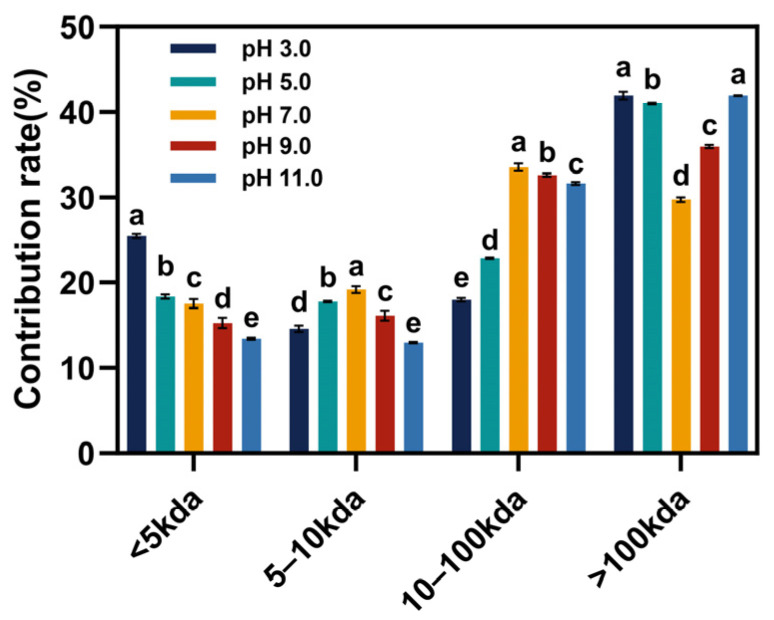
Relative molecular weight distribution of EGP at pH 3.0, 5.0, 7.0, 9.0, and 11.0. Note: Distinct letters denote significant differences among samples (*p* < 0.05).

The polydisperse nature of the molecular weight distribution of EGP was analyzed, as illustrated in Figure 1 and Table 1. The size exclusion chromatography (SEC) of EGP under different pH treatments revealed a broad molecular weight distribution ranging from 152 kDa to 5.7 kDa. Furthermore, other small molecules (<5 kDa), which likely corresponded to peptides or even free amino acids, were also identified [29]. The molecular weight distribution of EGP was categorized into four components for discussion purposes: below 5 kDa, 5–10 kDa, 10–100 kDa, and above 100 kDa. With the increase in pH, the concentration of components with a MW below 5 kDa exhibited a gradual decrease, whereas that of components with a MW between 5 and 10 kDa initially increased and then decreased. The predominant proportion of components with a molecular weight ranging from 10 to 100 kDa was observed at pH 7.0, with the majority of the MW sizes being concentrated around 32 kDa. Additionally, the proportion of components with a MW greater than 100 kDa significantly increased compared with pH 7.0 after undergoing pH treatment, and the predominant MW sizes concentrated around 125 kDa. A chromatographic peak at 152 kDa was observed at pH 5.0, which is possibly attributed to protein denaturation and aggregation near the isoelectric point.

### 3.2. Amino Acid Composition

The amino acid composition of EGP is presented in Table 2, revealing the presence of 17 different amino acids. The essential amino acids of EGP account for a significant proportion (51.51%) of the total amino acid content, surpassing that found in soybean protein isolate (SPI) (39.33%) [16]. The ten essential amino acids were threonine (45.70 mg/g), cystine (27.93 mg/g), valine (52.45 mg/g), methionine (28.14 mg/g), isoleucine (34.40 mg/g), leucine (63.66 mg/g), tyrosine (47.42 mg/g), phenylalanine (45.04 mg/g), histidine (33.21 mg/g), and lysine (56.09 mg/g), which indicates that EGP is a rich source of ideal essential amino acids. EGP was also characterized by a high content of hydrophobic amino acids (364.78 mg/g), including glycine (40.12 mg/g), alanine (50.82 mg/g), valine (52.45 mg/g), methionine (28.14 mg/g), isoleucine (34.40 mg/g), leucine (63.66 mg/g), phenylalanine (45.04 mg/g), and proline (50.14 mg/g). The abundance of hydrophobic amino acids contributes to the enhanced surface hydrophobicity of EGP. The HAA content in EGP exceeds that in tree peony (*Paeonia suffruticosa* Andr.) seed protein (302.00 mg/g) and is lower than that in *spirulina* protein hydrolysate (420.00 mg/g) [30,31]. The ratio of aromatic amino acids to branched-chain amino acids in EGP (61.44%) exceeds that in SPI (49.72%). This suggests that EGP can contribute significantly towards enhancing muscle metabolism and maintaining protein homeostasis [32].

The EAA composition of EGP was relatively well balanced, with most of the EAA content meeting and exceeding the WHO/FAO requirements for children. However, certain components, such as tyrosine (47.42 mg/g), lysine (56.09 mg/g), and leucine (63.66 mg/g), did not meet the WHO/FAO requirements for children but did meet the adult amino acid content standards. The amino acid analysis results indicate that EGP possesses an optimal amino acid composition, suggesting its potential to serve as a valuable dietary protein in nutritional food supplements.

### 3.3. Fourier Transform Infrared Spectroscopic Analysis (FTIR)

**Figure 2:**
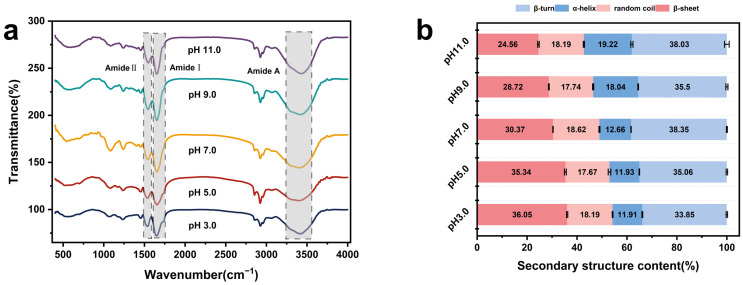
(**a**) Fourier transform infrared spectroscopy (FTIR) spectra of EGP at pH 3.0, 5.0, 7.0, 9.0, and 11.0. (**b**) Secondary structure of EGP at pH 3.0, 5.0, 7.0, 9.0, and 11.0.

The FTIR spectrum can reveal the structural alterations of proteins at the levels of secondary structure and hydrogen bonding force. Figure 2a shows the FTIR spectra of EGP at pH 3.0, 5.0, 7.0, 9.0, and 11.0 in the wavelength range of 4000–400 cm^−1^. The characteristic absorption peaks observed in the amide A region (3500–3000 cm^−1^) correspond to O-H stretching vibrations mainly caused by water or proteins, which are primarily associated with hydrogen bonding within the polypeptide backbone [33]. The changes observed in the characteristic peaks of the amide A region may be attributed to the disruption or degradation of hydrogen bonds between different protein groups in an acidic or alkaline environment, leading to alterations in the characteristic absorption peaks of O-H stretching vibrations. The spectra of all EGP samples subjected to different pH treatments exhibited characteristic peaks at 1510–1580 cm^−1^ (amide II region, N-H bending, and C=N stretching) and 1600–1700 cm^−1^ (amide I region and C=O stretching vibration), which correspond to the main characteristic peaks of proteins [34]. The two transmittance peaks (the amide I region and the amide II region) exhibited a significant decrease following pH treatment, particularly under acidic conditions. These alterations suggest that the pH treatment induced the rearrangement of peptide side chains or changes in the secondary structure of proteins.

The difference in FTIR spectra under pH treatment confirmed the transition of the secondary structure. The complex coupled vibration of amide II limits its use for secondary structure characterization, making the amide I bands of proteins the most important peaks for revealing the protein’s secondary structure [35]. The pH treatment significantly altered the distribution of α-helices, β-sheets, β-turns, and random coils in EGP (Figure 2b and Table 3). The ratio of β-sheet content in EGP at pH 3.0–5.0 (35.34–36.05%) was notably higher than that at pH 9.0–11.0 (24.56–28.72%). Conversely, the ratio of α-helix content in EGP at pH 3.0–5.0 (11.91–11.93%) was notably lower than that at pH 9.0–11.0 (18.04–19.22%). The acidic pH treatments of EGP were observed to increase the β-sheet structure content and decrease that of α-helix structures. α-Helices are primarily stabilized by intramolecular hydrogen bonding between the carbonyl oxygen (-CO) and (NH-) hydrogen within the peptide chain [36], while β-sheets are associated with intermolecular hydrogen bonding between protein molecule peptide chains [37]. The higher β-sheet structure content in an acidic environment may have been due to the breakdown of the hydrogen bonding structure and the increase in aggregate content [38], which is consistent with the subsequent particle size distribution and also accounts for the lower solubility of EGP under acidic conditions. The random coil content of EGP at pH 3.0, 5.0, 7.0, 9.0, and 11.0 did not change significantly, with the lowest proportion being observed at pH 5.0 due to decreased electrostatic repulsion leading to protein aggregation near the isoelectric point. Compared with acidic conditions, alkaline environments had a greater altering effect on the secondary structure content of EGP.

### 3.4. Foaming Properties

**Figure 3:**
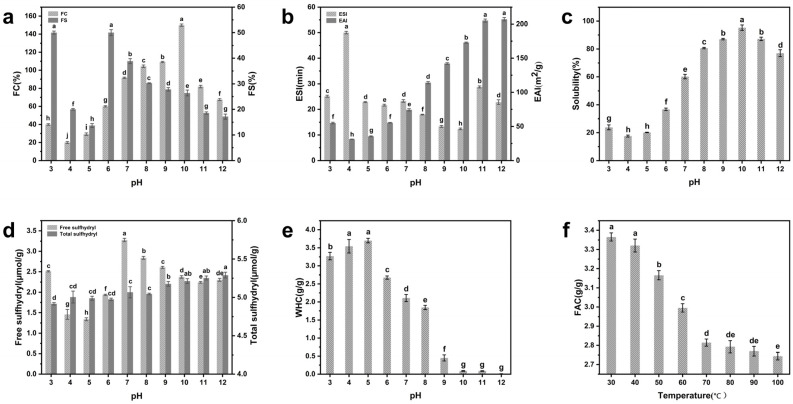
(**a**) Foaming properties, (**b**) emulsifying properties, (**c**) solubility, (**d**) free sulfhydryl and total sulfhydryl, and (**e**) water-holding capacity of EGP in different acidic and alkaline environments. (**f**) Fat absorption capacity of EGP at different temperatures. Note: Distinct letters denote significant differences among samples (*p* < 0.05).

In addition to excellent nutritional properties, most proteins also exhibit strong surface activity, enabling them to provide exceptional foaming characteristics. Foaming capacity (FC) refers to the interfacial area generated during the foaming process, while foam stability (FS) pertains to its ability to stabilize bubbles. As shown in Figure 3a, the FC rates of EGP at pH 3.0–12.0 were 40.00%, 20.00%, 29.20%, 60.00%, 91.60%, 104.40%, 109.20%, 150.00%, 82.00%, and 67.60%, respectively. The high EGP FC at pH values far from the isoelectric point may be attributed to the unfolding of protein molecules and enhanced protein–water interaction, which facilitate air envelopment and create favorable conditions for bubble formation [39]. The solubility of a substance is also a crucial factor for its foaming properties [40]. The foam capacity results of EGP were generally in line with the solubility curve, and higher FC may be correlated with greater solubility. The FS of EGP reached its peak at pH 6.0; the high FS of EGP in a near-neutral environment was attributed to its high hydrophobic amino acid content, which enhanced protein interaction, formed cohesive interfacial membranes, and stabilized the air bubbles [39]. However, as the pH value further increased, there was a gradual decline in FS, dropping from 50.00% at pH 6.0 to 17.16% at pH 12.0. A similar result was reported by Bing et al. [41], where lentinus edodes protein also showed a similar trend. The explanation may be that fully extended EGP molecules at a pH far from the isoelectric point possess a greater number of net surface charges, leading to enhanced interaction with water. Interaction among protein molecules is concomitantly attenuated, forming weaker interfacial membranes, ultimately reducing the FS of EGP.

The maximum FC of EGP exceeds that of peanut protein (64.8% ± 0.2%) and soybean protein (65.7 ± 0.5%) [11], indicating the potential suitability of EGP as a foaming agent in food applications.

### 3.5. Emulsifying Properties

Emulsifying properties refer to the capability to facilitate the formation and maintenance of protein emulsion and encompass the emulsion activity index (EAI) and the emulsion stability index (ESI).

The results are shown in Figure 3b and indicate that the EAI of EGP reached its minimum near the isoelectric point and increased significantly as the pH increased from 4.0 to 12.0, reaching a maximum of 207.09 m^2^/g. Chen et al. [11] also observed that the minimum EAI for *Chlorella pyrenoidosa* protein (CPP) and *Arthospira platensis* protein (APP) occurred near the isoelectric point, with a tendency to increase with higher pH in alkaline environments. The low solubility of EGP at its isoelectric point hinders oil–water interface migration and reduces emulsion activity, while its high solubility in alkaline environments promotes the interaction between the oil phase and the aqueous phase, thereby enhancing its emulsifying activity. The EAI of EGP at neutral pH (74.25 m^2^/g) is lower than that of *Haematococcus pluvialis* soluble proteins (80 ± 1 m^2^/g) but higher than SPI (32.57 m^2^/g) and rice residue protein (10.00 m^2^/g) [16,29,42]. Therefore, EGP shows potential as an alternative emulsifying agent in the food industry.

Figure 3b shows that the ESI of EGP at pH 4.0 is greater than that at any other pH level. This finding aligns with the research findings of Chen et al. [11], who demonstrated that *Chlorella pyrenoidosa* (CPP) and *Arthospira platensis* (APP) exhibit high ESIs at the isoelectric point. EGP exhibited low ESIs at pH 9.0 and 10.0; the decrease in the ESI may be attributed to the alkaline environment, which restricts the interaction among protein molecules and leads to the formation of weak interface membranes [43]. In summary, the pH treatment affected the solubility and surface charge of EGP, leading to significant changes in its emulsifying properties.

### 3.6. Solubility

As shown in Figure 3c, the solubility of EGP reached a minimum of 17.54% near the isoelectric point. Similar findings have been reported for various food proteins, including soy protein and mung bean protein, and in other microalgal proteins, such as *Haematococcus pluvialis* protein [9,34,44]. The minimum solubility near the isoelectric point may be due to the fact that the positive and negative charges of proteins are in a relative equilibrium state, leading to decreased electrostatic repulsion and reduced solubility [14]. The increase in pH from 4.0 to 10.0 resulted in an increase in solubility from 17.54% to 95.28%, respectively. This may be attributed to the heightened negative charge on the surface among protein molecules under alkaline conditions, leading proteins to disperse easily in deionized water due to increased electrostatic repulsion [45]. The solubility of EGP at pH 7.0 (60.16%) was superior to that of SPI (58.00%) (Chee et al. [16]).

### 3.7. Free Sulfhydryl and Total Sulfhydryl

Free sulfhydryl groups and total sulfhydryl groups in proteins are closely related to the structural alterations of protein molecules [19]. As shown in Figure 3d, the content of free sulfhydryl was the lowest at pH 5.0 (1.34 μmol/g) and the highest at pH 7.0 (3.28 μmol/g). However, within the pH range of 7.0–12.0, the free sulfhydryl groups in EGP exhibited a decreasing trend as the pH increased, which can be attributed to their propensity to form disulfide bonds when exposed to a polar environment for an extended period, leading to a reduction in SH clusters [46]. The variation in the total sulfhydryl group content in EGP remained relatively stable compared to that of the free sulfhydryl group. The total sulfhydryl content of EGP slightly increased with the rise in pH, possibly due to a reduction in disulfide bonds in EGP under alkaline conditions compared to acidic conditions [19], leading to an upward trend in the total sulfhydryl content of EGP. Furthermore, the content of free sulfhydryl in EGP was generally higher in alkaline environments compared with acidic ones. These data indicate that extreme environments far from the isoelectric point promote EGP molecule unfolding and degradation, exposing sulfhydryl groups [47]. The minimum content of free sulfhydryl near the isoelectric point may be explained by the burying of free sulfhydryl due to protein particle aggregation. This finding aligns with the analytical data on the average particle size.

### 3.8. Water-Holding Capacity

Water-holding capacity (WHC) is indicative of a protein–water interaction and contributes to moisture retention in food. Figure 3e illustrates the relationship between pH and WHC in EGP. As the pH increased from 3.0 to 5.0, the WHC of EGP increased slightly from 3.27 g/g to 3.70 g/g and then rapidly decreased from 3.70 g/g to 0.09 g/g with the increase in pH from 5.0 to 10.0, respectively, after which the lowest WHC was maintained in the strongly alkaline environment (0.01–0.09 g/g). There was no direct correlation between protein solubility and WHC, indicating that high protein solubility does not necessarily indicate high WHC, which is consistent with other studies [48]. The present higher WHC result can be attributed to lower solubility and lower α-helix structure content [11]. These factors promote protein hydration and, thus, explain the high WHC of EGP in acidic environments. The low WHC of EGP in strongly alkaline environments may be attributed to the unfolding of the protein molecules, resulting in a flexible protein structure that fails to retain water when binding it [38]. The WHC of EGP at neutral pH (2.11 g/g) is comparable to or even higher than that of some microalgal proteins according to previous studies, such as *Kappaphycus alvarezii* (2.22 ± 0.04 g/g), *E. compressa* (1.53 ± 0.07 g/g), and *E. Tobeulosa* (1.32 ± 0.11 g/g) [49].

Overall, the WHC of EGP falls within the range of requirements for sticky foods (1.49–4.72 g/g), indicating that EGP is a suitable option for sticky and bakery goods [50].

### 3.9. Fat Absorption Capacity

Fat absorption capacity (FAC) indicates the degree to which protein side chains bind to fats [51]. Figure 3f shows that as the temperature increased from 30 °C to 70 °C, the FAC of EGP decreased from 3.37 g/g to 2.81 g/g, respectively. Furthermore, with a further increase in temperature from 70 °C to 100 °C, there was a slight decrease in the FAC of EGP from 2.81 g/g to 2.74 g/g. The low FAC of EGP at high temperatures may be attributed to the disruption of intramolecular and intermolecular hydrogen bonds, as well as the reduction in hydrophobic points. This leads to the disintegration of the ordered structure of EGP and the reduction in its lipid absorption capacity. The FAC of EGP (2.74–3.37 g/g) is greater than those of wheat germ protein (1.04–1.32 g/g) and oat bran protein (1.51–2.96 g/g) [52]. Therefore, EGP is a potential candidate for high-fat products, such as emulsion and bakery products.

### 3.10. Particle Size Distribution and Zeta Potential

**Figure 4:**
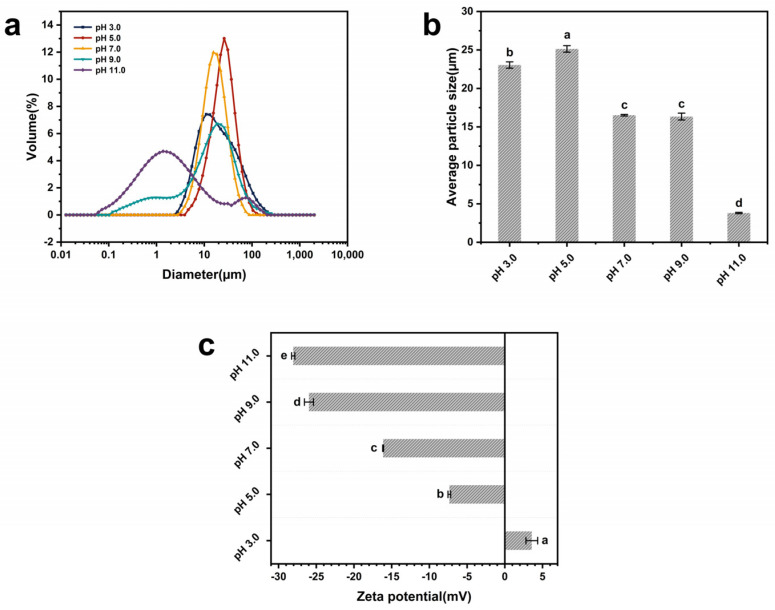
(**a**) Particle size distribution, (**b**) average particle size, and (**c**) zeta potential of EGP at pH 3.0, 5.0, 7.0, 9.0, and 11.0. Note: Distinct letters denote significant differences among samples (*p* < 0.05).

The particle size distribution of the EGP dispersions at pH 3.0, 5.0, 7.0, 9.0, and 11.0 was analyzed to investigate the protein aggregation extent and average particle size (Figure 4a,b and Table 4). The average particle size of EGP varied from 3.81 μm to 25.13 μm in the pH range 3–11. EGP showed unimodal particle distribution at pH 3.0, 5.0, 7.0, and 9.0, with average particle sizes of 23.04 µm, 25.13 µm, 16.52 µm, and 16.34 µm, respectively. At pH 11.0, EGP presented a bimodal particle distribution with peak areas of 88.9% (1.33 µm) and 11.1% (55.32 µm), and its average particle size was 3.81 µm. The results of the EGP particle size distribution align with previous findings regarding the effect of pH treatment on amaranth (*Amaranthus hychondriacus*) seed protein isolates’ particle size distribution [53]. The average particle size of EGP in acidic environments was larger than in alkaline environments. In particular, EGP had the largest average particle size (25.13 µm) near the isoelectric point, which may be attributed to the low net surface charge near the isoelectric point, leading to protein particle aggregation and forming large irreversible protein aggregates [54]. The reduction in average particle size of EGP at pH 11.0 may be attributed to the unfolding of protein molecules in an alkaline environment, leading to an increase in net surface charge, which hinders the formation of protein aggregates and promotes more uniform dispersion of proteins in deionized water [55].

The stability of a colloidal dispersion can be reflected by the zeta potential [56]. The zeta potentials of EGP at pH 3.0, 5.0, 7.0, 9.0, and 11.0 were 3.58 mV, −7.36 mV, −16.14 mV, −25.98 mV, and −28.06 mV, respectively (Figure 4c and Table 4). The high zeta potential in alkaline environments indicates an increase in the surface net charge and electrostatic repulsion of EGP, leading to a more uniform dispersion of protein molecules in water. Additionally, the reason why EGP has higher FC in alkaline environments may be that proteins with higher net surface charge are more easily adsorbed at the interface [55]. The net surface charge of EGP at pH 3.0 and 5.0 was lower compared with that in neutral and alkaline environments. Reduced net surface charge under acidic conditions disrupts the electrostatic repulsion among protein molecules, leading to significant aggregation and larger particle distribution [57], which aligns with the obtained particle size distribution results. The comparison of the resulting zeta potential with the obtained solubility data suggests that higher electrostatic repulsion and increased ionic hydration contribute to the enhanced solubility of EGP in alkaline environments.

### 3.11. Exogenous Fluorescence Spectroscopy

**Figure 5:**
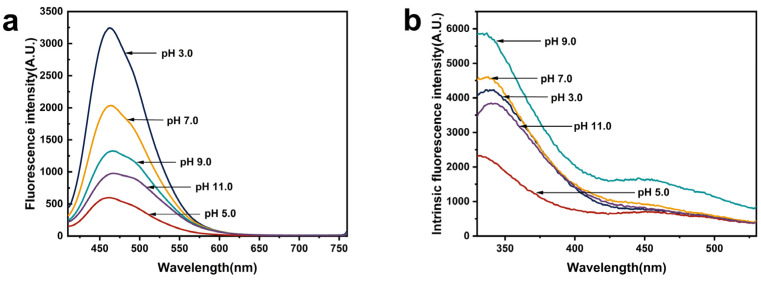
(**a**) Exogenous and (**b**) intrinsic fluorescence spectroscopy results of EGP at pH 3.0, 5.0, 7.0, 9.0, and 11.0.

Surface hydrophobicity is closely associated with the conformation, stability, and functional properties of proteins and is used to characterize the hydrophobic groups present on the protein surface. The peak value can be utilized as an indicator of hydrophobic intensity, a high value of which is correlated with higher fluorescence intensity [25]. The surface hydrophobicity of EGP under different pH treatments is shown in Figure 5a and Table 4, with values of 3244.67, 598.77, 2039.67, 1326.67, and 977.70 A.U. at pH 3.0, pH 5.0, pH 7.0, pH 9.0, and pH 11.0, respectively. The value at pH 3.0 exhibited significantly higher levels compared with other pH conditions, potentially due to the exposure of more hydrophobic regions as a result of protein subunit dissociation at this pH. The minimum value at pH 5.0 might be attributed to the precipitation and aggregation of protein particles near the isoelectric point, leading to the concealment of hydrophobic groups within the protein aggregates [58], which is consistent with the results of the particle size distribution.

### 3.12. Intrinsic Fluorescence Spectroscopy

Intrinsic fluorescence is generated by aromatic amino acid residues and characterizes the conformational changes in proteins, particularly alterations in the tertiary structure [26]. As shown in Figure 5b, the λmax and fluorescence intensity values of EGP at pH 3.0, 5.0, 7.0, 9.0, and 11.0 were 337.00 nm, 332.00 nm, 338.00 nm, 337.00 nm, and 340 nm and 4229 A.U., 2325 A.U., 4608, A.U., 5875 A.U., and 3852 A.U., respectively. The data indicate a reduction in fluorescence intensity and a slight red shift in λmax to 340 nm at pH 11.0, attributed to the extremely alkaline environment among protein molecules, which increases electrostatic repulsion and consequently facilitates the dissociation of polypeptide segments and the unfolding of proteins [59]. This is consistent with the results of high electrostatic repulsion indicated by the zeta potential of EGP at pH 11.0. The lowest values for both λmax and fluorescence intensity were observed at pH 5.0. The quenching and blue shift in intrinsic fluorescence may be attributed to the formation of EGP aggregate structures near the isoelectric point, leading to the burial of exposed aromatic amino acids and subsequent reduction in fluorescence intensity. Similarly, Chen et al. [11] reported that *Arthospira platensis* protein (APP) had the lowest fluorescence intensity at the isoelectric point. After pH treatment, the fluorescence intensity of EGP exhibited significant changes, indicating that the acidic and alkaline environment had an impact on the tertiary structure of EGP.

### 3.13. X-ray Diffraction Analysis

**Figure 6:**
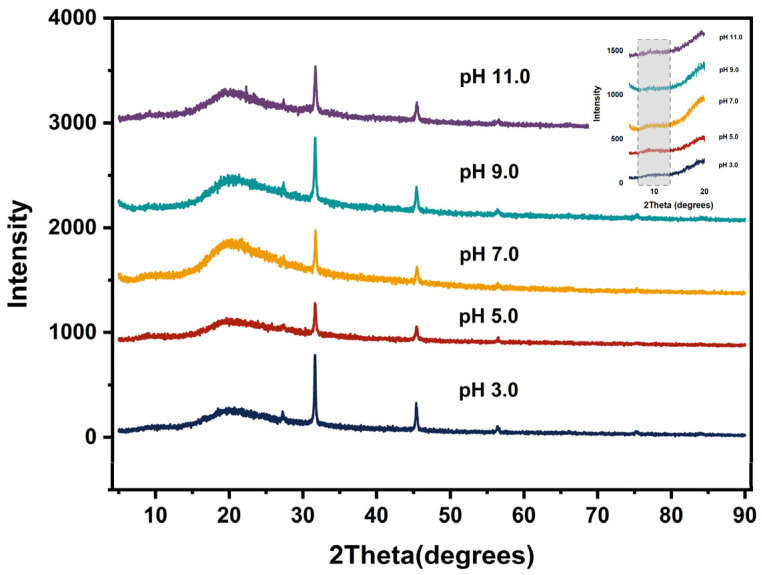
XRD patterns of EGP at pH 3.0, 5.0, 7.0, 9.0, and 11.0.

XRD diffraction was utilized to evaluate the amorphous or crystal structure of biopolymers. The XRD spectra of EGP at pH 3.0, 4.0, 7.0, 9.0, and 11.0 are shown in Figure 6. The diffraction patterns revealed sharp and narrow diffraction peaks at 31° and 45°, indicative of well-defined crystalline regions. Based on the previous literature studies and standard X-ray diffraction patterns, the peak attribution analysis shows that sharp and narrow diffraction peaks at 31° and 45° may be due to the existence of NaCl [60]. In addition, broad peaks at 9° and 20° were detected simultaneously, and similar peaks were also observed in alfalfa protein isolates by Sahni et al. [61]. The diffraction peaks obtained at approximately 9° and 20° correspond to α-helix and β-sheet secondary structures, respectively (Chen et al. [62]). The peak intensities at 9° and 20° in EGP after pH treatment were both decreased compared with pH 7.0, which may be attributed to alterations in the protein secondary structure. Das et al. [45] reported similar results for soybean meal protein under different pH treatments. The intensity of the diffraction peak is correlated with the crystal size and crystallinity of the protein, where the higher the diffraction peak intensity, the higher the crystal size and crystallinity in the protein [63]. The pH treatments induced changes in diffraction peak intensity, reflecting alterations in the crystal size and crystallinity of EGP. The decreases in crystal size and crystallinity after acidic pH treatment can be attributed to the reorganization in the crystal structure of protein molecules caused by their denaturation.

### 3.14. Thermal Properties

**Figure 7:**
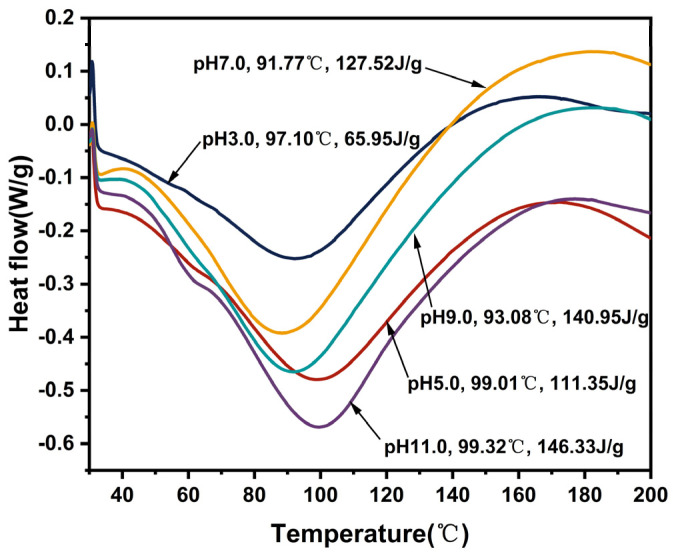
Differential scanning calorimetry (DSC) thermographs of EGP at pH 3.0, 5.0, 7.0, 9.0, and 11.0.

Differential scanning calorimetry (DSC) is utilized for the analysis of thermal properties of protein isolates and the assessment of thermal behavior and stability in other food systems. The assessment of thermal stability primarily relies on parameters such as onset temperature (T_o_), denaturation temperature (T_d_), end temperature (T_e_), and enthalpy (ΔH). A single peak usually indicates that a protein isolate is composed of one protein or several substances with similar thermal stability [53]. Performing the evaluation based on only the denaturation temperature or enthalpy value may provide limited insights into the thermal stability of proteins; thus, combined analyses are necessary for accurate assessment. The thermal properties of EGP obtained under different pH treatments are illustrated in Figure 7 and Table 5. The denaturation temperatures of EGP at pH 3.0, 5.0, 7.0, 9.0, and 11.0 were 97.10 °C, 99.01 °C, 91.77 °C, 93.08 °C, and 99.32 °C, respectively. The corresponding enthalpy values were 65.95 J/g, 111.35 J/g, 127.52 J/g, 140.95 J/g, and 146.33 J/g, respectively.

These data indicate EGP had high T_d_ and high ΔH at pH 11.0; this may be attributed to heightened thermal stability, which enhances protein resistance to high temperatures and promotes increased synergies between alkaline environment and heat during the process [64]. Furthermore, the elevated thermal stability may be associated with enhanced hydrophobic linkages among protein molecules in alkaline environments. The denaturation temperatures of EGP at pH 3.0 and 5.0 exceed those at pH 7.0 and 9.0 due to the formation of protein aggregates requiring higher temperatures to denature proteins. The denaturation temperature of EGP is similar to that of other plant and animal proteins, such as glycinin (89.70 °C; Mo et al. [65]) and hemp seed protein (95.00 °C; Tang et al. [66]) but higher than that of broccoli leaf protein (66.06 °C; Rawiwan et al. [67]). The denaturation temperature of various proteins is also influenced by diverse extraction methods, protein purity, water content, and other contributing factors [28].

## 4. Conclusions

In this study, we demonstrated that different acidic and alkaline environments significantly impact the structural characteristics and functional properties of EGP. According to our findings, EGP is rich in hydrophobic and essential amino acids. Its molecular weight distribution exhibits polydispersity, with sizes being predominantly concentrated around 32 kDa and 125 kDa. In our experiments, following treatment in different acidic and alkaline environments, there was a significant increase in components with a molecular weight exceeding 100 kDa. EGP exhibited a high α-helix content and a low β-sheet content in alkaline environments, leading to enhanced protein hydration and resulting in high WHC. The changes in alkaline environments had a greater impact on the alteration in the protein secondary structure compared with acidic environments. The weak surface net charge near the isoelectric point resulted in reduced electrostatic repulsion, leading to the aggregation and precipitation of large particles with low solubility, free sulfhydryl, and surface hydrophobicity. The total sulfhydryl group content exhibited minimal changes and continued to demonstrate an upward trend as the pH value increased. The reduced FAC at elevated temperatures was attributed to the increased exposure of hydrophobic points to high-temperature environments. The higher zeta potential in alkaline environments indicated greater electrostatic repulsion force, which was reflected in the high solubility, FC, and EAI in the alkaline environments. The tertiary structure of EGP also underwent changes, resulting in a blue shift in λmax and the lowest fluorescence intensity values being observed at pH 5.0. The XRD patterns indicated that EGP had well-defined crystalline regions due to the presence of NaCl. The pH treatment led to a significant decrease in crystal size and crystallinity, particularly in acidic environments. The denaturation temperature and enthalpy of EGP at pH 11.0 were significantly high, and the denaturation temperatures at pH 3.0 and 5.0 were higher than those at pH 7.0 and 9.0 due to protein aggregation. EGP demonstrated superior functionality in different acidic and alkaline environments, indicating that it could serve as a valuable functional ingredient in diverse food applications.

## Figures and Tables

**Table 1:** Relative molecular weight distribution of EGP at pH 3.0, 5.0, 7.0, 9.0 and 11.0.

Sample	Molecular Weight Distribution (%)			
	>100 kDa	10–100 kDa	5–10 kDa	<5 kDa
pH 3.0	41.95 ± 0.46 ^a^	17.97 ± 0.20 ^e^	14.61 ± 0.38 ^d^	25.47 ± 0.26 ^a^
pH 5.0	41.03 ± 0.08 ^b^	22.85 ± 0.09 ^d^	17.77 ± 0.09 ^b^	18.35 ± 0.24 ^b^
pH 7.0	29.75 ± 0.28 ^d^	33.53 ± 0.44 ^a^	19.18 ± 0.40 ^a^	17.54 ± 0.52 ^c^
pH 9.0	35.95 ± 0.20 ^c^	32.61 ± 0.19 ^b^	16.15 ± 0.57 ^c^	15.29 ± 0.59 ^d^
pH 11.0	41.94 ± 0.50 ^a^	31.65 ± 0.17 ^c^	12.97 ± 0.08 ^e^	13.44 ± 0.11 ^e^

Note: Values were means ± standard deviation. Distinct letters within the same columns denote significant differences among samples (*p* < 0.05).

**Table 2:** Total amino acids in EGP.

Amino Acid	EGP (mg/g)	FAO/WHO Reference for Children (mg/g)	FAO/WHO Reference for Adults (mg/g)
Asparagine (Asp)	76.81 ± 0.07	-	-
Threonine (Thr)	45.70 ± 0.03	14	9
Serine (Ser)	44.24 ± 0.18	-	-
Glutarnine (Glu)	87.82 ± 0.19	-	-
Glycine (Gly)	40.12 ± 0.03	-	-
Alaine (Ala)	50.82 ± 0.11	-	-
Cystine (Cys)	27.93 ± 0.04	25	17
Valine (Val)	52.45 ± 0.05	35	13
Methionine (Met)	28.14 ± 0.12	-	-
Isoleucine (Ile)	34.40 ± 0.27	28	13
Leucine (Leu)	63.66 ± 0.14	66	19
Tyrosine (Tyr)	47.42 ± 0.08	63	19
Phenylalanine (Phe)	45.04 ± 0.19	-	-
Histidine (His)	33.21 ± 0.17	19	-
Lysine (Lys)	56.09 ± 0.05	58	16
Arginine (Arg)	58.69 ± 0.16	-	-
Proline (Pro)	50.14 ± 0.11	-	-
Essential amino acids	434.06 ± 0.27	-	-
Non-essential amino acids	408.65 ± 0.76	-	-
Hydrophobic amino acids	364.78 ± 0.30	-	-
Hydrophilic amino acids	165.30 ± 0.29	-	-
Acidic amino acids	164.63 ± 0.23	-	-
Basic amino acids	148.00 ± 0.29	-	-
Aromatic amino acids	92.47 ± 0.12	-	-
Branched-chain amino acids	150.51 ± 0.32	-	-
Negatively charged amino acids	254.57 ± 0.36	-	-
Positively charged amino acids	148.00 ± 0.29	-	-

Note: Results are presented as means ± standard deviation (*n* = 3).

**Table 3:** Secondary structure of EGP at pH 3.0, 5.0, 7.0, 9.0, and 11.0.

Sample	β-Sheets (%)	α-Helices (%)	β-Turns (%)	Random Coils (%)
pH 3.0	36.05 ± 0.25 ^a^	11.91 ± 0.17 ^d^	33.85 ± 0.28 ^c^	18.19 ± 0.15 ^ab^
pH 5.0	35.34 ± 0.37 ^b^	11.93 ± 0.11 ^d^	35.06 ± 0.30 ^b^	17.67 ± 0.53 ^b^
pH 7.0	30.37 ± 0.07 ^c^	12.66 ± 0.06 ^c^	38.35 ± 0.19 ^a^	18.62 ± 0.20 ^a^
pH 9.0	28.72 ± 0.16 ^d^	18.04 ± 0.13 ^b^	35.50 ± 0.42 ^b^	17.74 ± 0.21 ^b^
pH 11.0	24.56 ± 0.31 ^e^	19.22 ± 0.46 ^a^	38.03 ± 0.93 ^a^	18.19 ± 0.23 ^ab^

Note: Values are means ± standard deviation. Distinct letters within the same columns denote significant differences among samples (*p* < 0.05).

**Table 4:** Average particle size, zeta potential, and surface hydrophobicity of EGP at pH 3.0, 5.0, 7.0, 9.0, and 11.0.

Samples	Average Particle Size (µm)	Zeta Potential (mV)	Hydrophobicity
pH = 3.0	23.04 ± 0.41 ^b^	3.58 ± 0.78 ^a^	3244.67 ± 16.01 ^a^
pH = 5.0	25.13 ± 0.42 ^a^	−7.36 ± 0.18 ^b^	598.77 ± 7.95 ^e^
pH = 7.0	16.52 ± 0.10 ^c^	−16.14 ± 0.06 ^c^	2039.67 ± 37.45 ^b^
pH = 9.0	16.34 ± 0.45 ^c^	−25.98 ± 0.60 ^d^	1326.67 ± 10.50 ^c^
pH = 11.0	3.81 ± 0.08 ^d^	−28.06 ± 0.22 ^e^	977.70 ± 8.45 ^d^

Note: Values are means ± standard deviation. Distinct letters within the same columns denote significant differences among samples (*p* < 0.05).

**Table 5:** Onset temperature (T_o_), denaturation temperature (T_d_), endset temperature (T_e_), and enthalpy (ΔH) of EGP at pH 3.0, 5.0, 7.0, 9.0, and 11.0.

Sample	T_o_ (°C)	T_d_ (°C)	T_e_ (°C)	ΔH (J/g)
pH 3.0	55.45 ± 0.42 ^a^	97.10 ± 0.28 ^b^	136.82 ± 0.34 ^d^	65.95 ± 0.16 ^e^
pH 5.0	53.31 ± 0.36 ^a^	99.01 ± 0.14 ^a^	149.55 ± 0.21 ^a^	111.35 ± 0.25 ^d^
pH 7.0	51.36 ± 0.38 ^b^	91.77 ± 0.23 ^d^	145.91 ± 0.12 ^b^	127.52 ± 0.47 ^c^
pH 9.0	49.82 ± 0.12 ^c^	93.08 ± 0.47 ^c^	143.89 ± 0.65 ^c^	140.95 ± 0.34 ^b^
pH 11.0	46.94 ± 0.43 ^d^	99.32 ± 0.31 ^a^	145.33 ± 0.23 ^b^	146.33 ± 0.37 ^a^

Note: Values are means ± standard deviation. Distinct letters within the same columns denote significant differences among samples (*p* < 0.05).

## Data Availability

The original contributions presented in the study are included in the article, further inquiries can be directed to the corresponding author.
